# The Counteracting Effect of Chrysin on Dietary Fructose-Induced Metabolic-Associated Fatty Liver Disease (MAFLD) in Rats with a Focus on Glucose and Lipid Metabolism

**DOI:** 10.3390/molecules30020380

**Published:** 2025-01-17

**Authors:** Gabriela Campanher, Nelson Andrade, Joanne Lopes, Cláudia Silva, Maria João Pena, Ilda Rodrigues, Fátima Martel

**Affiliations:** 1 Unit of Biochemistry, Department of Biomedicine, Faculty of Medicine of Porto, University of Porto, 4200-319 Porto, Portugal; gabi.campanher@hotmail.com (G.C.); nandrade@med.up.pt (N.A.); claudiasilva@med.up.pt (C.S.); mariajoao0502@gmail.com (M.J.P.); irodrigues@med.up.pt (I.R.); 2 School of Medical Sciences, University of Örebro, Campus USÖ, S-701 82 Örebro, Sweden; 3 REQUIMTE/LAQV, Department of Chemical Sciences, Faculty of Pharmacy, University of Porto, R. J. Viterbo Ferreira, 228, 4050-313 Porto, Portugal; 4 Department of Pathology, Faculty of Medicine of Porto, University of Porto, 4200-319 Porto, Portugal; joannedlopes@msn.com; 5 Instituto de Investigação e Inovação em Saúde (i3S), University of Porto, 4200-135 Porto, Portugal

**Keywords:** fructose, chrysin, liver, adipose tissue, tissue injury

## Abstract

The prevalence of metabolic syndrome has been exponentially increasing in recent decades. Thus, there is an increasing need for affordable and natural interventions for this disorder. We explored the effect of chrysin, a dietary polyphenol, on hepatic lipid and glycogen accumulation, metabolic dysfunction-associated fatty liver disease (MAFLD) activity score and oxidative stress and on hepatic and adipose tissue metabolism in rats presenting metabolic syndrome-associated conditions. Rats fed a chow diet were separated into four groups: Control (tap water), Fructose (tap water with 10% fructose), Chrysin (tap water+ chrysin (100 mg/kg body weight/d)), and Fructose + Chrysin (tap water with 10% fructose + chrysin (100 mg/kg body weight/d, daily)) (for 18 weeks). When associated with the chow diet, chrysin reduced hepatic lipid and glycogen storage, increased the hepatic antioxidant potential of glutathione and reduced de novo lipogenesis in the adipose tissue. When associated with the high fructose-diet, chrysin attenuated the increase in lipid and glycogen hepatic storage, improved the MAFLD activity score, decreased hepatic lipid peroxidation, increased the antioxidant potential of glutathione, and improved lipid and glucose metabolic markers in the liver and adipose tissue. In conclusion, our results suggest that chrysin is a beneficial addition to a daily diet for improvement of hepatic metabolic health, particularly for individuals suffering from metabolic syndrome.

## 1. Introduction

Metabolic syndrome (MetS) consists of a group of metabolic abnormalities (abdominal obesity, high blood pressure, high blood sugar, high serum triglycerides, and low serum high-density lipoproteins) that increases the risk for cardiovascular diseases, type 2 diabetes, metabolic dysfunction-associated fatty liver disease (MAFLD), and cancer [1]. MetS is reaching an epidemic proportion in developed countries and is a major contributor to premature morbidity and mortality [2]. Fructose consumption has dramatically increased in the past few decades (e.g., in the form of high fructose corn syrup, a common sweetener used in the food industry), and the intake of fructose-containing foods and drinks is epidemiological and experimentally linked to MetS. Indeed, this sugar is unique in its remarkable ability to induce MetS in rodents [3], and epidemiological works suggest a strong correlation between high fructose intake and obesity, MAFLD, type 2 diabetes, kidney dysfunction, and cardiovascular disease [4]. As a consequence, new interventions to reduce the metabolic consequences of high-fructose feeding are urgently needed.

Chrysin (5,7-dihydroxyflavone) is one of the major flavonoids found in plants belonging to the genera *Oroxylum*, *Chamomille*, and *Passiflora* and is also abundant in honey and propolis [5]. This dietary polyphenol exhibits many biological activities, including cardioprotective, anti-inflammatory, anticancer, and antidiabetic activities [5].

Our group recently verified that chrysin was able to decrease fructose uptake and the expression of fructose transporters in human intestinal epithelial cells in vitro [6]. This led us to test the possible beneficial effect of chrysin on MetS induced by fructose-feeding. In agreement with the expected, we verified that rats fed fructose (10% in drinking water) for 18 weeks exhibit numerous features of MetS (including hypertension, hyperinsulinemia, hyperleptinemia, hypertriglyceridemia, higher angiotensin II blood levels, and higher hepatic triacylglycerides and fibrosis levels and liver: body ratio) and chrysin was able to revert some of the MetS characteristics induced by fructose-feeding, namely the hypertension, hepatic fibrosis, hyperinsulinemia, and the increase in angiotensin II serum levels [7]. These effects were observed despite no alterations in body weight and global energy intake were found (Figure S1). Moreover, at the intestinal level, chrysin was able to interfere with fructose-induced changes in intestinal permeability, villous morphology and microbiota [8], and altered intestinal permeability and dysbiosis associate with MetS [9]. The anti-MetS properties of chrysin in high-fructose fed rat models were later confirmed by others [10,11].

It is well established that excess fructose consumption has negative effects at the hepatic level, including increased fatty acid production (fatty liver), increased oxidative stress, and insulin resistance. These effects have been linked to MAFLD, progression to non-alcoholic steatohepatitis (NASH), and hepatocellular carcinoma (HCC) [12,13]. Thus, the rise in MAFLD/NASH cases progressing to HCC in developed countries may be attributed to increased consumption of fructose [12,13]. Despite this, common physiological and biochemical parameters that confirm the efficacy of high-fructose feeding in inducing MetS in rat models include confirmation of hyperglycemia, hypertension, obesity, dyslipidemia and hyperinsulinemia/insulin resistance [3,14,15], and the consequences of excessive fructose feeding at the hepatic level have been, in comparison, much less studied [14].

For this reason, the aim of the present study was to explore the potential ameliorating effects of chrysin on changes in hepatic lipid levels, glycogen storage, MAFLD activity (NAS) score, and oxidative stress markers and on the hepatic and adipose tissue expression of genes associated with carbohydrate and lipid metabolism brought forth by a high-fructose diet (HFD) in rats.

## 2. Results

### 2.1. Liver Lipid Accumulation

**Figure 1:**
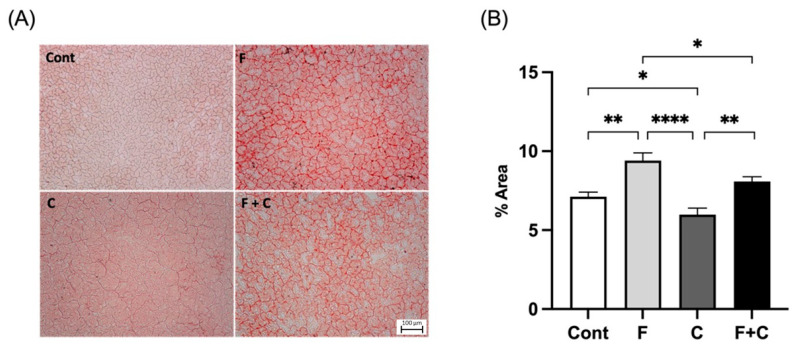
(**A**) Representative pictures of histological liver samples stained with Oil red O from Control (Cont), Fructose (F), Chrysin (C), and Fructose + Chrysin (F + C) groups. Magnification 200×. Scale bar: 100 μm. (**B**) Lipid content in % of total area measured in liver samples from Cont, F, C, and F + C groups. Results are expressed as arithmetic means ± SEM (*n* = 6 in each group). Significant *p*-values are marked with * (*p* < 0.05), ** (*p* < 0.01), **** (*p* < 0.0001) (one-way ANOVA with Tukey’s multiple comparison).

In order to evaluate the capacity of chrysin to attenuate lipid accumulation in the liver in both control and HFD conditions, liver tissues from all four groups were stained with Oil red O (Figure 1A). Analysis of the results showed that the hepatic lipid content of Fructose (F) group was significantly higher in comparison to all other groups (Figure 1B). Moreover, the lipid content in the Chrysin (C) and Fructose + Chrysin (F + C) animals were significantly lower than in Control (Cont) and F animals, respectively, but there was no statistically significant difference between Cont and F + C animals (Figure 1B). This clearly shows that chrysin significantly lowers hepatic lipid accumulation under both a standard diet and a HFD context.

### 2.2. Liver Glycogen Storage

**Figure 2:**
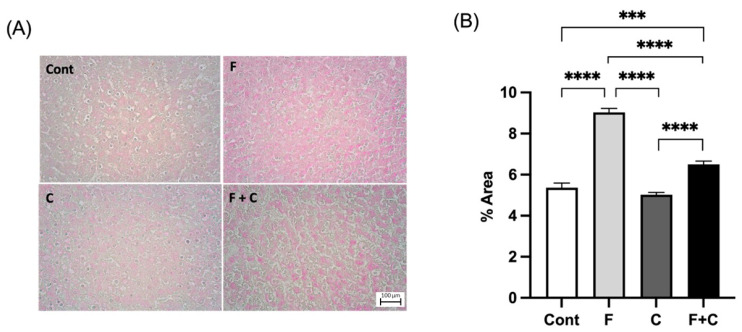
(**A**) Representative pictures of histological liver samples stained with PAS from Control (Cont), Fructose (F), Chrysin (C), and Fructose + Chrysin (F + C) groups. Magnification 200×. Scale bar: 100 μm. (**B**) Glycogen content in % of total area measured in liver samples from Cont, F, C, and F + C groups. Results are expressed as arithmetic means ± SEM (*n* = 6 in each group). Significant *p*-values are marked with *** (*p* < 0.001), **** (*p* < 0.0001) (one-way ANOVA with Tukey’s multiple comparison).

For examination of the potential effect of chrysin on liver glycogen storage in both control and HFD conditions, Periodic Acid–Schiff (PAS) staining was performed (Figure 2A) and a quantitative comparison of glycogen content between groups was made (Figure 2B). The glycogen content in the liver of F animals was significantly higher when compared to all the other groups. Moreover, liver glycogen content in F + C animals was significantly lower than in F animals (Figure 2B). This clearly shows that chrysin was able to ameliorate hepatic glycogen in a HFD context.

### 2.3. NAS Score

**Figure 3:**
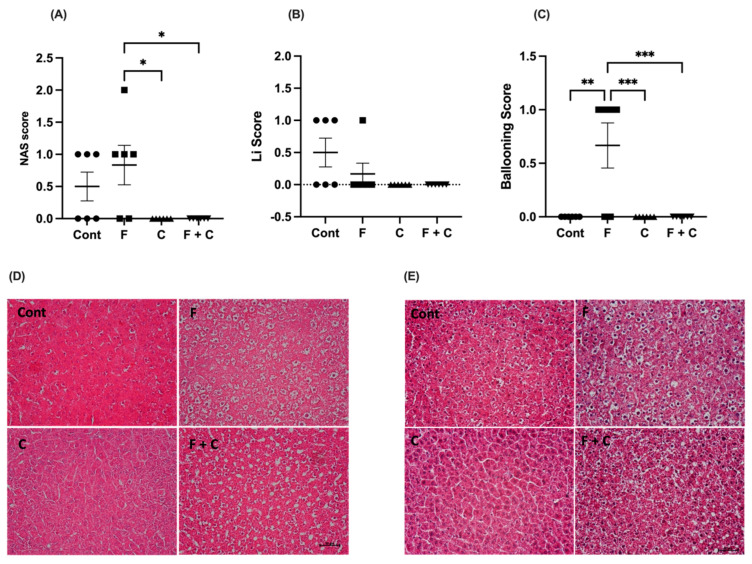
(**A**) NAS score; (**B**) Lobular inflammation (Li) score; (**C**) Hepatocyte ballooning score in Control (Cont), Fructose (F), Chrysin (C), and Fructose + Chrysin (F + C) groups. Results are expressed as arithmetic means ± SEM (*n* = 6 in each group). Significant *p*-values are marked with * (*p* < 0.05), ** (*p* < 0.01), *** (*p* < 0.001) (one-way ANOVA with Tukey’s multiple comparison). The NAS score was calculated as the sum of steatosis grade (0–3), lobular inflammation (0–2), and hepatocyte ballooning (0–2). (**D**) Representative pictures of histological liver samples colored with CAB staining. (**E**) Representative pictures of histological liver samples stained with H&E coloration. Magnification 200×. Scale bar: 100 μm.

The sectioned liver tissues from all animals were stained with either Hematoxylin and Eosin (H&E) coloration or a Chromotrope Aneline Blue (CAB) staining (Figure 3D and Figure 3E, respectively). Both histological stainings were blindly evaluated by an experienced pathologist and given an NAS score, and the tissue degrees of steatosis, lobular inflammation, and hepatocyte ballooning were assessed.

The MAFLD activity score (NAS score) of Cont animals had no significant difference from any other group. Also, no difference was observed between C and F + C groups either, both receiving a combined score of 0 (no MAFLD). However, F rats received a significantly higher score as compared to both C and F + C groups (Figure 3A). So, we can conclude that chrysin was able to reduce the hepatic NAS score in a HFD situation.

In contrast, there was no difference in the lobular inflammation (LI) score between the groups (Figure 3B).

Finally, an assessment of ballooning revealed that ballooning was observed in F samples only (Figure 3C) and that the F group had a significantly higher degree of hepatocyte ballooning as compared to all other groups. The remaining groups did not differ from each other (Figure 3C). No degree of fibrosis or steatosis (S) was observed in any of the animals. Hence, chrysin shows the capacity to improve NAS scores when introduced to a HFD, particularly in terms of hepatocyte ballooning.

### 2.4. Liver Oxidative Stress Markers

To evaluate oxidative stress levels in the liver samples, MDA levels and glutathione levels were determined in the animals from the four groups.

**Figure 4:**
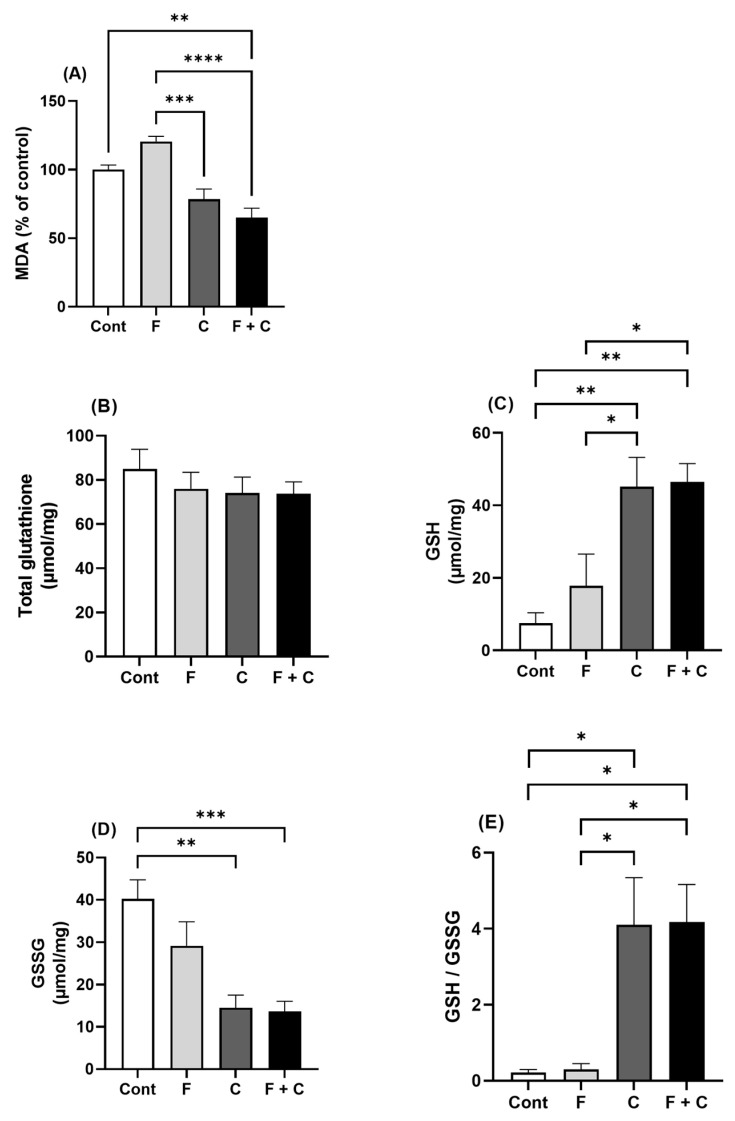
Hepatic levels of oxidative stress markers. (**A**) MDA (malondialdehyde); (**B**) Total glutathione; (**C**) GSH (reduced glutathione); (**D**) GSSG (oxidized glutathione); (**E**) and GSH/GSSG ratio, in Control (Cont), Fructose (F), Chrysin (C), and Fructose + Chrysin (F + C) groups. Results are expressed as arithmetic means ± SEM (*n* = 5–6/group). Significant *p*-values are marked with * (*p* < 0.05), ** (*p* < 0.005), *** (*p* < 0.001), **** (*p* < 0.0001) (one-way ANOVA with Tukey’s multiple comparison).

MDA levels reflect the extent of lipid peroxidation. As shown in Figure 4A, a significant reduction was found in the F + C group vs. both the Cont and F groups. So, chrysin appears to be able to completely abolish the effects mediated by HFD on MDA levels, even for values lower than those in the Control situation.

Glutathione is the principal intracellular antioxidant buffer against oxidative stress and mainly exists in the forms of reduced glutathione (GSH) and oxidized glutathione (GSSG). As shown in Figure 4B, there were no differences in total glutathione levels between all groups of animals. However, a significant increase in the concentrations of GSH in C and F + C groups of animals was found, when compared to the Cont and the F groups, respectively (Figure 4C). This was accompanied by a significant decrease in hepatic levels of GSSG in the rats from C and F + C groups, as compared to the Cont group (Figure 4D). As a result, an increase in GSH/GSSG ratio, in relation to both Cont and F groups, was observed in the F + C group (Figure 4E) So, it is clear that chrysin has a beneficial effect on hepatic glutathione status (by reducing GSSG levels and increasing GSH levels). Importantly, this effect is not affected by HFD.

### 2.5. Expression of Hepatic Lipid Metabolic Markers

**Figure 5:**
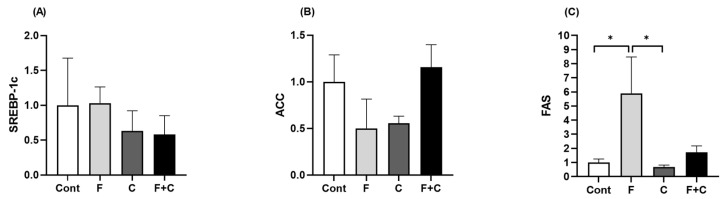
Liver mRNA expression of (**A**) *SREBP-1c*, (**B**) *ACC*, and (**C**) *FAS*, measured in Control (Cont), Fructose (F), Chrysin (C), and Fructose + Chrysin (F + C) groups. Results are expressed as arithmetic means ± SEM (*n* = 4–6/group). Expression was calculated using 2^−ΔCT^ with significant *p*-values marked with * (*p* < 0.05) (one-way ANOVA with Tukey’s multiple comparison).

As a means of evaluating the effect of chrysin on hepatic lipid metabolism, RT-qPCR was performed in order to quantify the gene expression levels of sterol regulatory element-binding protein 1c (*SREBP-1c*), acyl-CoA carboxylase (*ACC*), and fatty acid synthase (*FAS*). No significant differences in *SREBP-1c* and *ACC* mRNA levels were observed between any of the groups (Figure 5A,B). However, *FAS* expression was significantly increased in the Fructose group, when compared to both Cont and C groups. This increase was no longer significant with the addition of Chrysin to the HFD (Figure 5C). So, chrysin is able to abolish the rise in *FAS* expression induced by the HFD.

### 2.6. Expression of Hepatic Glucose Metabolic Markers

**Figure 6:**
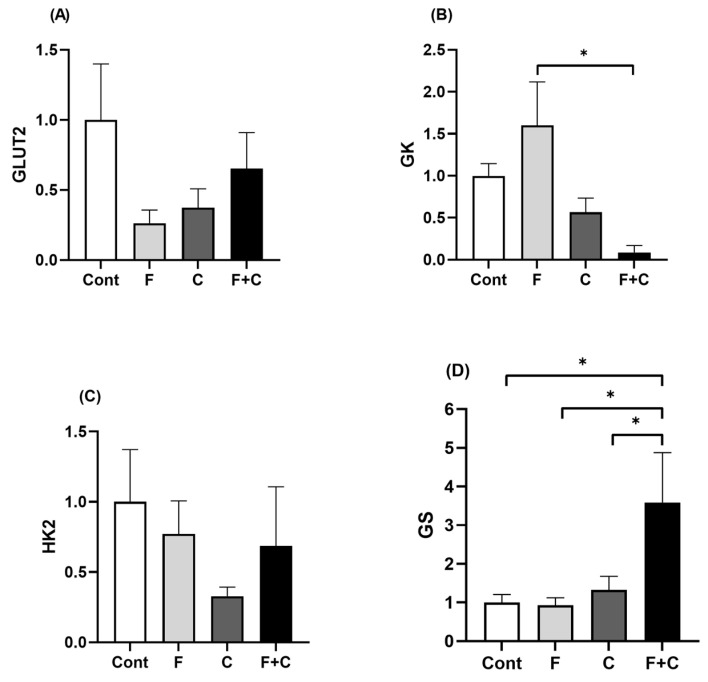
Liver mRNA expression of (**A**) *GLUT2*, (**B**) *GK*, (**C**) *HK2*, and (**D**) *GS*, measured in Control (Cont), Fructose (F), Chrysin (C), and Fructose + Chrysin (F + C) groups. Results are expressed as arithmetic means ± SEM (*n* = 4–6/group). Expression was calculated using 2^−ΔCT^ with significant *p*-values marked with * (*p* < 0.05) (one-way ANOVA with Tukey’s multiple comparison).

In order to assess the potential effect of chrysin treatment on hepatic glucose metabolism, the expression levels of glucose transporter 2 (*GLUT2*), glucokinase (*GK*), hexokinase II (*HK2*), and glycogen synthetase (*GS*) were measured using RT-qPCR. No significant differences in *GLUT2* and *HK2* mRNA levels were observed between the groups (Figure 6A,C). In contrast, there was a significant decrease in *GK* expression in the F + C group as compared to the F group. However, no such change was observed when chrysin was introduced to the control diet (Figure 6B). Moreover, *GS* expression was significantly higher in F + C as compared to all other groups, with the remaining groups not differing from each other (Figure 6D). These results point to a significant decline in *GK* expression and to an increase in *GS* expression promoted by chrysin upon a HFD.

### 2.7. Expression of Genes Related to Lipid Metabolism in Adipose Tissue

**Figure 7:**
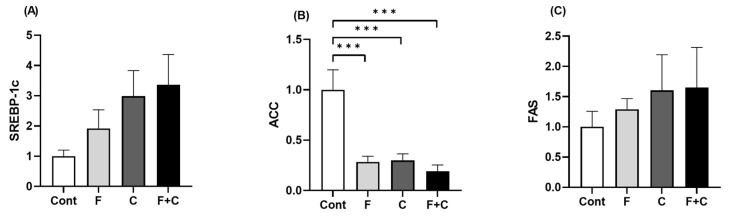
Adipose tissue mRNA expression of (**A**) *SREBP-1c*, (**B**) *ACC*, and (**C**) *FAS*, measured in Control (Cont), Fructose (F), Chrysin (C), and Fructose + Chrysin (F + C) groups. Results are expressed as arithmetic means ± SEM (*n* = 5–6/group). Expression was calculated using 2^−ΔCT^ with significant *p*-values marked with *** (*p* < 0.001) (one-way ANOVA with Tukey’s multiple comparison).

Exploration of the effects of fructose and/or chrysin on lipid metabolism was extended to the adipose tissue, by assessing the expression levels of *SREBP-1c*, *ACC*, and *FAS* by RT-qPCR. No significant differences in *SREBP-1c* and *FAS* expression were observed (Figure 7A,C). However, a much higher expression of *ACC* was measured in the Cont as compared to all other groups, which were not significantly different from each other (Figure 7B). This result shows that both Fructose and Chrysin are able to decrease adipose tissue *ACC* expression when associated with a healthy diet.

### 2.8. Expression of Genes Related to Glucose Metabolism in Adipose Tissue

**Figure 8:**
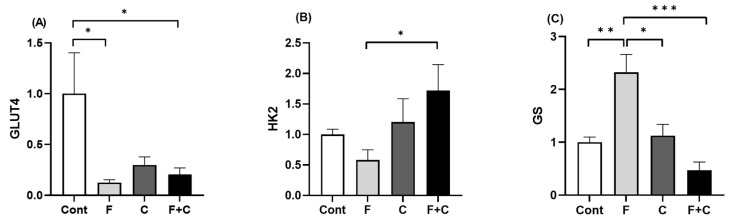
Adipose tissue mRNA expression levels of (**A**) *GLUT4*, (**B**) *HK2*, and (**C**) *GS*, measured in Control (Cont), Fructose (F), Chrysin (C), and Fructose + Chrysin (F + C) groups. Results are expressed as arithmetic means ± SEM (*n* = 6/group). Expression was calculated using 2^−ΔCT^ with significant *p*-values marked with * (*p* < 0.05), ** (*p* < 0.01), *** (*p* < 0.001).

Lastly, expression of genes related to glucose metabolism in the adipose tissue was investigated in order to determine the ability of chrysin to improve glucose metabolic markers in this tissue. For that purpose, *GLUT4*, *HK2*, and *GS* mRNA expression levels were measured using RT-qPCR. The expression levels of *GLUT4* were significantly decreased in the F group as compared to Cont, even in the presence of Chrysin (Figure 8A). In contrast, the expression of *HK2* was significantly increased in F + C as compared to the F group (Figure 8B). Finally, the expression of *GS* was significantly increased in the F group when compared to Cont, but interestingly, this effect disappeared in the F + C group (Figure 8C). This indicates that chrysin is able to counteract Fructose-induced increase in *GS* gene expression, but not Fructose-induced decrease in *GLUT4* expression. Moreover, chrysin increases the expression of *HK2* when associated with a HFD.

## 3. Discussion

Fructose-feeding constitutes a validated experimental model for MetS induction in rodents, although the efficacy of this model depends on the rodent species, gender, study duration and evidently the amount of fructose added to the diet [3,13]. Confirmation of the induction of MetS in this model is commonly established by assessing parameters such as hyperglycemia, hypertension, obesity, dyslipidemia, and hyperinsulinemia/insulin resistance [3,14,15]. The consequences of excessive fructose feeding at the hepatic level in this model have been, in comparison, much less studied [14], although fructose consumption in humans is linked to MAFLD [16]. MAFLD is a manifestation of MetS affecting about one-third of the population worldwide and has progressive pathological potential for liver cirrhosis and HCC through NASH [17]. In our previous published work, we verified that fructose-feeding was able to induce changes at the hepatic level, namely an increase in liver/body weight ratio, hepatic fibrosis and triacylglycerides (TAG) content, and that chrysin was able to revert the increase in hepatic fibrosis levels induced by the HFD [7].

In the present study, we aimed to further explore the potential MeS-ameliorating properties of chrysin at the hepatic level, both in the context of a healthy diet and a HFD. MAFLD is intimately linked to hepatic steatosis, inflammation, insulin resistance (IR), oxidative stress (OS), and ballooning. So, we decided to focus on evaluation of hepatic lipid and glycogen accumulation, MAFLD activity score (NAS score) (which reflects the combined degree of steatosis, lobular inflammation, and hepatocyte ballooning), oxidative stress markers, and carbohydrate and lipid metabolic markers. Overall, the results point to a significant improvement in hepatic health following chrysin supplementation.

In terms of hepatic lipid accumulation, a significant decrease in lipid content was observed when chrysin was added to both diets. This reduced hepatic steatosis could be attributed to a reduction in de novo lipogenesis promoted by chrysin, as gene analysis showed that the marked increase in *FAS* expression prompted by a HFD was no longer present upon chrysin treatment. *FAS* is an enzyme involved in de novo hepatic lipogenesis and possesses regulatory properties [18]. It is upregulated in a state of overconsumption, particularly associated with high-fructose intake, resulting in increased lipid storage [19], and this is confirmed by our results. The decline in *FAS* expression associated with chrysin treatment thus certainly contributes to the reduction in hepatic lipid accumulation observed in the histological analysis. *ACC* and *SREBP-1c* mRNA levels, however, were not significantly changed by chrysin intake on either diet. *ACC* enables the conversion of acetyl-CoA into malonyl-CoA, and it is a rate-limiting enzyme in de novo lipogenesis [20], whereas *SREBP-1c* is a transcription factor that regulates cholesterol and fatty acid synthesis [21]. Both promote obesity and MAFLD [22]. Not only did chrysin have no effect on the expression of either, but neither did the HFD as compared to the healthy controls. This could be due to the fact that we used a lower amount of fructose in the water (10%) when compared to other HFD studies, since the expression of both *ACC* and *SREBP-1c* were expected to rise following a HFD [23,24].

Reduced liver lipid accumulation is desirable for all individuals but is particularly significant for MetS patients. Accumulation of lipids in the liver is predominantly observed in the form of lipid droplets causing hepatocyte ballooning, which is identified by its *Mallory–Denk* bodies containing cytoplasm [25]. It is associated with lipotoxicity of the tissue and promotes hepatocyte death [26]. Evaluation of the NAS score did identify an increase in hepatocyte ballooning associated with a HFD, but interestingly this increase was abolished by chrysin. These results allow us to relate the reduced accumulation of lipids with an improvement in hepatocyte function observed in the animals treated with chrysin on a HFD. It is worth to mention that in the present study, hepatic lipid levels were evaluated by measuring total lipid storage, and no investigation was performed regarding individual lipids, namely cholesterol, cholesteryl esters and TAG. In this same animal model, we verified that the HFD-induced increase in hepatic TAG was not improved by chrysin [7]. This observation suggests that chrysin specifically affects hepatic cholesterol/cholesteryl ester levels. Nevertheless, a more detailed picture of the hepatic lipid composition would aid in further understanding the specific improvement in hepatic lipid composition promoted by chrysin.

Hepatocyte ballooning is likewise associated with excessive glycogen accumulation in hepatocytes [26], and excessive glycogenosis is present in MAFLD patients [26,27]. Assessment of hepatic glycogen storage in this study likewise showed a beneficial effect of chrysin implementation in the context of a HFD. Indeed, chrysin partially inhibited HFD-induced increase in hepatic glycogen levels. A reduction in hepatic glycogen accumulation is promoted by a restored balance between glycogen synthesis and breakdown. Further investigation of targeted genes showed a significant decrease in *GK* gene expression promoted by chrysin in a HFD. *GK* catalyzes the first crucial step of glycogen synthesis, which is the conversion of glucose into glucose-6-phosphate. Its overexpression is associated with a state of overconsumption in order to promote hepatic clearance of glucose from the portal vein [28]. The reduction in the expression of *GK* induced by chrysin, in the presence of a HFD, may indeed contribute to the reduction in glycogen storage observed in the same animals, implying this as a potential mechanism of hepatic glucose metabolic improvement.

Measurements of *HK2* and *GLUT2* expression levels did not show any significant improvements by chrysin treatment nor changes induced by a HFD. *HK2* also phosphorylates glucose into glucose-6-phosphate and its increased expression is associated with obesity. However, in the liver, *GK* is the predominant hexokinase expressed, providing approximately 95% of hepatocyte hexokinase activity [29] and so the absence of effect of fructose and chrysin upon *HK2* expression probably reflects its low relevance in that organ. Hepatic uptake of circulatory glucose is primarily carried out by *GLUT2*, a low-affinity membrane glucose transporter. Besides being the main hepatic glucose transporter, several carbohydrates such as fructose, galactose, and mannose are also transported by *GLUT2* [30]. Due to the rise in liver glucose output present in obese animals, GLUT2 expression was expected to rise in the HFD group [31,32]. However, in our study, neither HFD nor chrysin induced significant changes in *GLUT2* mRNA levels.

Hepatic *GS* expression was also measured, and a significant increase was found in the F + C group of animals. *GS* is a crucial enzyme in the conversion of glucose into glycogen, by catalyzing the addition of glucose molecules to the growing glycogen molecule. Although reduced hepatic *GS* activity has been demonstrated in patients with non-insulin dependent diabetes mellitus [33,34], evaluation of the effect of fructose-feeding on the expression of this enzyme in the rat liver is scarce and the results found either no effect [35,36,37] or an increase [38,39]. Our results are, importantly, very similar to two recent publications, where two other polyphenolic compounds were found to increase *GS* protein expression when associated with fructose feeding, although HFD *per si* did not change *GS* expression levels [35,36]. This effect of chrysin associated with fructose-feeding on *GS* may be interesting from the metabolic point of view, because this gene may be a candidate gene responsible for insulin resistance [40] and a recent study showed an improvement in glucose tolerance, appetite, and body weight following overexpression of *GS* in mice fed a high-fat diet [37].

Altogether, our results show that the increase in glycogen accumulation in fructose-fed animals is related to an increase in the *GK*-mediated conversion of glucose to glucose-6-phophate and unrelated to an increase in *GLUT2*-mediated glucose hepatic uptake or *GS*-mediated glycogen synthesis. Moreover, the results also show that the decrease in glycogen accumulation in F + C animals in relation to the F group is associated with a decrease in *GK* expression and occurs despite an increase in *GS* expression. This suggests that either *GS* activity does not reflect its expression levels, or that a marked increase in glycogen degradation counteracts the increased synthesis rates. These results also suggest that *GK* has an important regulatory role in hepatic glycogen metabolism, as the level of hepatic *GK* expression is a very good indicator of the overall effect of the treatments on hepatic glycogen stores. It should be noted that all the animals were sacrificed following an overnight fasting period, and this surely caused a decrease in glycogen storage levels. However, since all animals followed the same feeding pattern in preparation for the sacrifice, it is expected that glycogen storage in all the animals would be equally affected, thus not interfering with the differences between groups.

The adipose tissue is primarily an energy-storing reservoir but also performs important endocrine and metabolic roles with a significant influence on metabolic health. Preventing its dysfunction as a consequence of HFD/MetS is thus a desirable outcome. For this reason, we also explored the potential benefit of chrysin on the metabolic health of the adipose tissue, under both a healthy and a HFD. In relation to lipid metabolic enzyme markers, no significant changes in both *SREBP-1c* and *FAS* were found. In contrast, *ACC* expression was significantly decreased by both HFD and chrysin, both alone and when combined, when compared to the controls. The decrease in *ACC* expression in the HFD group was not expected, because *ACC* levels were shown to increase in the adipose tissue of obese women as compared to that of normal-weight women [41] and in the adipose tissue of rats fed a HFD [42], although no effect of HFD on *ACC* mRNA levels have also been described [43]. The effect of chrysin on *ACC* mRNA levels when this polyphenol was added to the chow diet (a significant decrease in *ACC* expression) may contribute to a beneficial effect of chrysin on lipid metabolism in the adipose tissue when associated with a healthy diet, by decreasing lipid storage in the adipose tissue. Nevertheless, it is important to mention that chrysin did not further reduce *ACC* expression levels when associated with the HFD.

In relation to glycogen, evaluation of targeted genes on adipose tissue painted a picture of how glycogen storage was potentially affected by HFD and chrysin in this tissue. HFD induced a significant rise in *GS* expression levels, which in turn was abolished by chrysin intake. Although the adipose tissue does not primarily store glycogen, its presence in adipocytes has been observed to increase in a state of food overconsumption/expansion of the tissue/obesity and tissue inflammation [44]. Hence, we can speculate that glycogen accumulation in the adipose tissue was attenuated by chrysin in a HFD via a reduction in *GS* expression. By limiting glycogen accumulation in adipose tissue, chrysin may contribute to the functional preservation of this tissue, helping to maintain metabolic health in spite of a HFD.

A previous study showed a reduction in adipose tissue expression of *HK2* in mice fed a HFD, resulting in repression of adipose tissue lipogenesis and promotion of non-esterified fatty acids release and stimulation of liver glucose production [45]. A decreased expression of *HK2* in obese and diabetic patients was also found and it was proposed that adipose tissue *HK2* loss contributes to the development of insulin resistance and consequently hyperglycemia [45]. Our current study does not observe a significant decrease in *HK2* expression upon a HFD but rather a significant augment in its expression following chrysin supplementation of a HFD.

In the present study, the expression of the insulin sensitive glucose transporter *GLUT4* in the adipose tissue was significantly decreased by HFD. A reduction in *GLUT4* expression and translocation, resulting in a reduction in the removal of systemic glucose, is associated with insulin-resistance and is an expected outcome of the HFD [46] implying its rise to be of interest. Consistent with this knowledge, hyperinsulinemia was observed in these animals [5]. However, we also observed that adding chrysin to the HFD did not attenuate this decline. A previous study on high fat and sucrose-induced diabetic rats found increased levels of skeletal muscle *GLUT4* upon chrysin treatment [47]. So, further exploration is necessary regarding the effects of chrysin on *GLUT4* expression due to contradictory results between studies.

The present study was exclusively performed in male rats; therefore, it is not necessarily translatable to both genders. A sex-dependent dimorphism has been observed in terms of adipose tissue distribution and composition [48]. Therefore, the results of the present study could differ significantly in a female population. Thus, further exploration on the effects of chrysin in females is needed in order to achieve a broader understanding applicable to the entire population.

Hepatic oxidative stress has been proposed as one of the mechanisms responsible for the adverse metabolic effects of fructose. Indeed, fructose feeding increases hepatic oxidative stress [49] which contributes to insulin resistance, hepatic steatosis, MAFLD, and NASH [50]. Elevation of lipid peroxidation, protein oxidation and levels of ROS such as superoxide anion free radical hydrogen peroxide are associated with fructose-feeding [51]. Moreover, reductions in antioxidant defense have also been reported in fructose-fed animals, including an altered ratio between reduced glutathione and oxidized glutathione (GSH/GSSG) [51]. Confirming the importance of oxidative stress in the pathogenesis of fructose-induced MetS, antioxidant therapy reduces oxidative stress in fructose-fed rats and results in beneficial effects. Of note, other dietary antioxidant compounds (e.g., resveratrol) were able to reverse fructose-induced insulin resistance [52] and hypertension [53].

In relation to the effects of chrysin on hepatic oxidative stress markers, we verified that chrysin completely abolished the increase in MDA levels caused by HFD. Moreover, it was even able to decrease MDA levels to values inferior to those of control animals. This proves the ability of chrysin to completely counteract the deleterious effects of HFD on lipid peroxidation. Moreover, chrysin was able to cause a significant increase in the antioxidant capacity of glutathione, as it reduced GSSG levels and increased GSH levels and GSH/GSSG ratio. Importantly, this beneficial effect on glutathione was maintained in the presence of HFD, thus proving the efficacy of the antioxidant properties of chrysin even in the presence of fructose. Besides the increase in the antioxidant capacity of glutathione that we observed and that was also described by others [54,55], the antioxidant effect of chrysin may also be related to an inhibition in ROS generation [54,56] and an upregulation of antioxidant enzymes such as catalase, superoxide dismutase [57], and heme oxygenase-1 [55].

The two previous studies evaluating the effect of chrysin upon high-fructose feeding rats concluded that chrysin, at the hepatic level, was able to decrease steatosis, ballooning and lobular inflammation, and showed antioxidant, anti-inflammatory, and antifibrotic effects [10,11]. This favorable effect of chrysin at the hepatic level was verified against higher levels of fructose-feeding (20–30% in the water) than those used in the present study. Considering that the concentration of fructose used in the present study is similar to that found in sugar-sweetened beverages [14], the present study thus suggests that chrysin is able to ameliorate the negative consequences of fructose doses frequently ingested by humans.

## 4. Materials and Methods

### 4.1. Animals

This study was carried out in 24 male CD Sprague Dawley rats aged 8 weeks old (270–320 g) from Charles River Laboratories (Chatillon/Chalaronne, France). Upon arrival, rats were housed in pairs, in an enriched environment, and maintained on a daily photoperiod of 12 h lighting schedule (20–22 °C) with free access to standard laboratory pellet food (total 2.67 kcal/g—carbohydrate 53.5%, fat 3%, and protein 18.5%; diet #4RF21 certificate, Mucedola, Milan, Italy) and tap water. Acclimatization took place for 10 days before starting the experimental protocol. All animal procedures were authorized by the Veterinary National Department of the Ministry of Agriculture, Rural Development and Fisheries (approval No. 20436/2022) and were performed in accordance with the Guidelines for Care and Use of Laboratory Animals of University of Porto. Handling and care of the animals were conducted in conformity with the European Community Council guidelines for the use of experimental animals (86/609/EEC) and Act 129/92. All experiments were approved by the Animal Ethics Committee of the Faculty of Medicine of Porto.

### 4.2. Experimental Design

MetS was induced in normoglycemic rats by replacing tap water with fructose 10% (*w*/*v*) in tap water. The 24 rats were randomly divided into four groups (6 rats/group), and subjected to the following treatments for 18 weeks:

(i) Control (Cont): rats in this group received standard diet and tap water and 0.5 mL of corn oil daily (added to food);
(ii) Fructose (F): rats in this group received standard diet and tap water supplemented with 10% (*w*/*v*) fructose and 0.5 mL of corn oil daily (added to food);
(iii) Chrysin (C): rats in this group received standard diet, tap water and a daily dose of chrysin (100 mg/kg body weight mixed in 0.5 mL of corn oil, added to food);
(iv) Fructose + Chrysin (F + C): rats in this group received standard diet, tap water supplemented with 10% (*w*/*v*) fructose and a daily dose of chrysin (100 mg/kg body weight mixed in 0.5 mL of corn oil, added to food).

All experimental groups were fed ad libitum with the standard laboratory chow diet during the experimental period. Fructose solution, corn oil in food, and chrysin in food were freshly prepared every day. Chrysin, d-fructose (99% purity) and corn oil were purchased from Sigma (St. Louis, MO, USA).

During the treatment period, body weight, glycemia, blood pressure, and heart rate were measured once a week. Food and beverage intake were monitored twice a week and energy ingestion was calculated. At the end of the 18-week treatment period and after an overnight fast period, the animals were sacrificed, blood was collected from the left ventricle, liver, heart, kidneys, pancreas, and epididymal adipose tissue were removed and weighed. Serum biochemical parameters and insulin, leptin, and angiotensin (Ang) II levels, liver triacylglycerides (TAG), and fibrosis levels were determined. All these parameters are reported in our previous publication [7]. The present results were obtained from liver and adipose tissue (epidydimal fat pad) that were extracted from all animals and immediately frozen with liquid nitrogen and stored at −80 °C.

### 4.3. Quantification of Hepatic Lipid Accumulation and Glycogen Content

The frozen liver tissues were embedded in Tissue-Tek and sectioned into 12 μm thick cryo-sections. They were then further transferred onto histological slides and stored at −80 °C. Two separate depths of the tissue were provided for all animals on each histological slide. Some of the hepatic tissue was fixated in 10% neutral-buffered formalin (10%) for 24 h, and then further embedded in paraffin, sectioned, and transferred into histological slides.

#### 4.3.1. Lipid Accumulation

As a means of evaluating hepatic lipid accumulation, the frozen liver samples were stained with Oil red O, which enables the visualization of neutral lipids and cholesterol esters in the tissue, following an optimized protocol [58]. The samples were photographed (Nikon ECLIPSE 50i, Nikon UK, Surbiton, UK) and 10 representative images chosen of each prepared slide (magnification: 200×). The images were further analyzed using ImageJ software version 2.14.0 (NIH, Bethesda, MD, USA) allowing for quantification of the total hepatic lipid content.

#### 4.3.2. Glycogen Content

Glycogen content was likewise assessed, using PAS staining of the frozen liver samples. Beginning with a 10 min incubation in periodic acid (10%), followed by a 5 min bath in running water, a submersion of the slides in Schiff solution for 30 min and once again a 10 min bath in running water. Then, a 30-s staining in hematoxylin and a final 10 min water bath was performed. The samples were photographed (Nikon ECLIPSE 50i, Surbiton, UK) and 5 representative images were chosen for each prepared slide (magnification: 200×). The images were further analyzed using ImageJ software version 2.14.0 (NIH, Bethesda, MD, USA) allowing for quantification of the total hepatic lipid content.

### 4.4. Determination of NAS Score

The NAS score was calculated in liver samples in order to determine the combined degree of steatosis (S), lobular inflammation (LI), and hepatocyte ballooning. Scores were given as follows: steatosis grade (0–3), LI (0–2) and hepatocyte ballooning (0–2). The NAS score (0–7) is the sum of all 3 scores [59]. The degree of fibrosis was likewise evaluated, however, not added to this particular score. The samples were blindly evaluated in cooperation with an experienced pathologist and two staining methods were used for assessment of the tissue. Both were performed on paraffin embedded liver tissue fixated in 10% neutral-buffered formalin (10%) for 24 h.

#### 4.4.1. H&E Staining

An H&E staining was used to evaluate the general tissue morphology, cell size, and distribution. All samples were primarily deparaffined and hydrated, following a 2 min staining with Mayers Hematoxylin. These were then rinsed for 15 min in running tap water and briefly submerged in distilled water and 95% ethanol. Counterstaining was performed with Eosin solution for additional 2 min. Samples were at last dehydrated in increasing alcohol concentrations ending in xylene and then mounted.

#### 4.4.2. CAB Staining

A CAB staining was used to visualize the degree of liver fibrosis. Post deparaffination and hydration, all samples were submerged in Celestine blue solution for 5 min and further stained in Gill’s hematoxylin for 5 additional min. Following a wash in running water and rinse in distilled water, a 5 min incubation in formobilic acid (10%) was succeeded by a staining in CAB solution for 10 min. Lastly, samples were dehydrated in increasing alcohol concentrations ending in xylene and then mounted.

### 4.5. Determination of Hepatic Oxidative Stress Markers (Malondialdehyde (MDA) and Glutathione Levels)

Hepatic levels of total and oxidized glutathione (GSSG) were determined using a glutathione colorimetric detection kit according to the manufacturer’s instructions (EIAGSHC, Thermo Fisher Scientific, Waltham, MA, USA). Reduced glutathione levels (GSH) were calculated according to the following reaction: Total Glutathione = GSH + 2 GSSG. Likewise, the MDA levels were tested with the aid of a commercial kit (MAK085, Sigma-Aldrich, Saint Louis, MO, USA).

### 4.6. RNA Extraction and Quantitative Real-Time PCR

Liver and adipose tissue extracted from all animals and immediately frozen with liquid nitrogen and stored at −80 °C were used. Both liver and adipose tissue were homogenized by grinding in liquid nitrogen, from which RNA was extracted with NZYOL (1 mL NZYOL:10 cm^2^ of tissue), followed by a chloroform phase separation (0.2 μL chloroform:1 mL NZYOL) and isopropanol precipitation (0.5 isopropanol: 1 NZYOL). Total RNA quality and quantity were evaluated on NanoDrop One (Thermo-Fisher Scientific, Lisbon, Portugal) at 230 nm, 260 nm and 280 nm.

Conversion of 1 µg total RNA into cDNA was performed using qScript cDNA SuperMix (Quanta Biosciences, Beverly, MA, USA), following the manufacturer instructions. For PCR, a master mix (MM) was prepared consisting of SYBRGreen Master Mix (Kapa Biosystems, Darmstadt, Germany), RNAse free-H_2_O, forward and reverse primers (100 mM stock, final concentration 10 mM; Table 1) at a 5:3:6:0.2 ratio. All samples were transferred into a 96-well plate and combined with the MM at a 1:9 ratio. Both adipose and hepatic gene expression were quantified through real-time quantitative PCR (qRT-PCR on a Lightcycler 96 (Roche, Porto, Portugal). Cycling conditions were as follows: denaturation (95 °C for 10 min), amplification, and quantification (95 °C for 10 s, annealing temperature for 10 s (Table 1) and 72 °C for 10 s, with a single fluorescence measurement at the end of the 72 °C for a 10 s segment) repeated for distinct number of cycles (Table 1) and a final melting step with a temperature ramp from 60 to 97 °C. Glyceraldehyde-3-phosphate dehydrogenase (GAPDH) was used as an endogenous control.

Gene expression was calculated with the formula 2^−ΔCq^, which represented the difference in the tested gene and the housekeeping gene Cq values. Values were compared against the control values.

### 4.7. Statistical Analysis

Statistical analysis was performed using GraphPad Prism version 8.0 (GraphPad software, Inc., La Jolla, CA, USA). One-way ANOVA was used for group comparisons followed by Tukey’s post hoc test to determine statistical significance. Statistical significance has been set at *p* < 0.05 with a 95% confidence interval.

## 5. Conclusions

In conclusion, chrysin promoted an improvement in hepatic health when introduced to a HFD in rats. Namely, it significantly limited the hepatic accumulation of glycogen and fats brought on by the HFD. Moreover, it improved the MAFLD activity (NAS) score, with a very evident effect on hepatocyte ballooning. It also prevented the increase in lipid peroxidation levels induced by the HFD and was able to increase the antioxidant potential of glutathione when associated with the HFD. Additionally, it aided in amelioration of specific markers of lipid and glucose metabolism, in both the liver (*FAS* and *GK*) and adipose tissue (*HK2* and *GS*). Regarding its implementation to a chow diet, it reduced hepatic lipid and glycogen storage, increased the antioxidant potential of glutathione in the liver and reduced de novo lipogenesis (*ACC*) in the adipose tissue. Ergo, the current results do promote the notion that chrysin is an effective therapeutic intervention for improvement of metabolic health. Further studies are necessary in order to explore additional metabolic benefits attributed to chrysin supplementation.

## Figures and Tables

**Table 1:** Primer sequences, annealing temperature (A_t_), and tissues used for gene analysis by qRT-PCR evaluation.

Gene	Primer Sequence (5′–3′)	A_t_ (°C)	Cycles	Tissue
*HK2*	Fwd: CAG CCT AGA CCA GAG CAT CC	59	60	Liver/Adipose Tissue
	Rev: CGC ATC TCT TCC ATG TAG CA			
*GS*	Fwd: AGA AAT CAC AGC CAT CGA GGC	60	50	Liver/Adipose Tissue
	Rev: GTT CAA GCC GTT TGG AGT CAC			
*GK*	Fwd: CAT ATG TGC TCC GCA GGA CTA G	60	50	Liver
	Rev: CTT GTA CAC GGA GCC ATC CA			
*FAS*	Fwd: GTG GGT CTC CTC CGA AGC CG	60	40	Liver/Adipose Tissue
	Rev: AGC ATG TCT TCG ATG TCG GTC AAG			
*ACC*	Fwd: CCT TGT CAA CGC ATG GGC GG	59	60	Liver/Adipose Tissue
	Rev: GGC TTT GGG GTG GGG AGT CG			
*SREBP-1c*	Fwd: GTG GGT CTC CTC CGA AGC CG	60	40	Liver/Adipose Tissue
	Rev: AGC ATG TCT TCG ATG TCG GTC AAG			
*GLUT2*	Fwd: TTC TGT GCC GTC TTC ATG TC	59	40	Liver
	Rev: TGG CCC AAT CTC AAA GAA AC			
*GLUT4*	Fwd: GGC CGG GAC ACT ATA CCC	58	50	Adipose Tissue
	Rev: CCC CAT CTT CAG AGC CGA T			
*GAPDH*	Fwd: GGC ATC GTG GAA GGG CTC ATG AC	70	45	Liver/Adipose Tissue
	Rev: ATG CCA GTG AGC TTC CCG TTA AGC			

Abbreviations: *HK2*, hexokinase 2; *GS*, glycogen synthetase; *GK*, glucokinase; *FAS*, fatty acid synthase; *ACC*, acyl-CoA carboxylase; *SREBP-1c*, sterol regulatory element-binding protein 1c; *GLUT2*, glucose transporter 2; *GLUT4*, glucose transporter 4; *GAPDH*, glyceraldehyde-3-phosphate dehydrogenase.

## Data Availability

The data presented in this study are available on request from the corresponding author.

## Supplementary Materials

The following supporting information can be downloaded at: https://www.mdpi.com/article/10.3390/molecules30020380/s1, Figure S1: Variation in rat body weight and energy intake (global, from food and from fluid) during the 18 weeks of treatment.
