# Supplementary material for: The Counteracting Effect of Chrysin on Dietary Fructose-Induced Metabolic-Associated Fatty Liver Disease (MAFLD) in Rats with a Focus on Glucose and Lipid Metabolism
Source: Molecules. 2025 Jan 17;30(2):380. doi: 10.3390/molecules30020380 (PMC11768066; doi:10.3390/molecules30020380)
Supplement: Supplementary file 1 [file molecules-30-00380-s001.zip › molecules-3395968-supplementary.pdf]

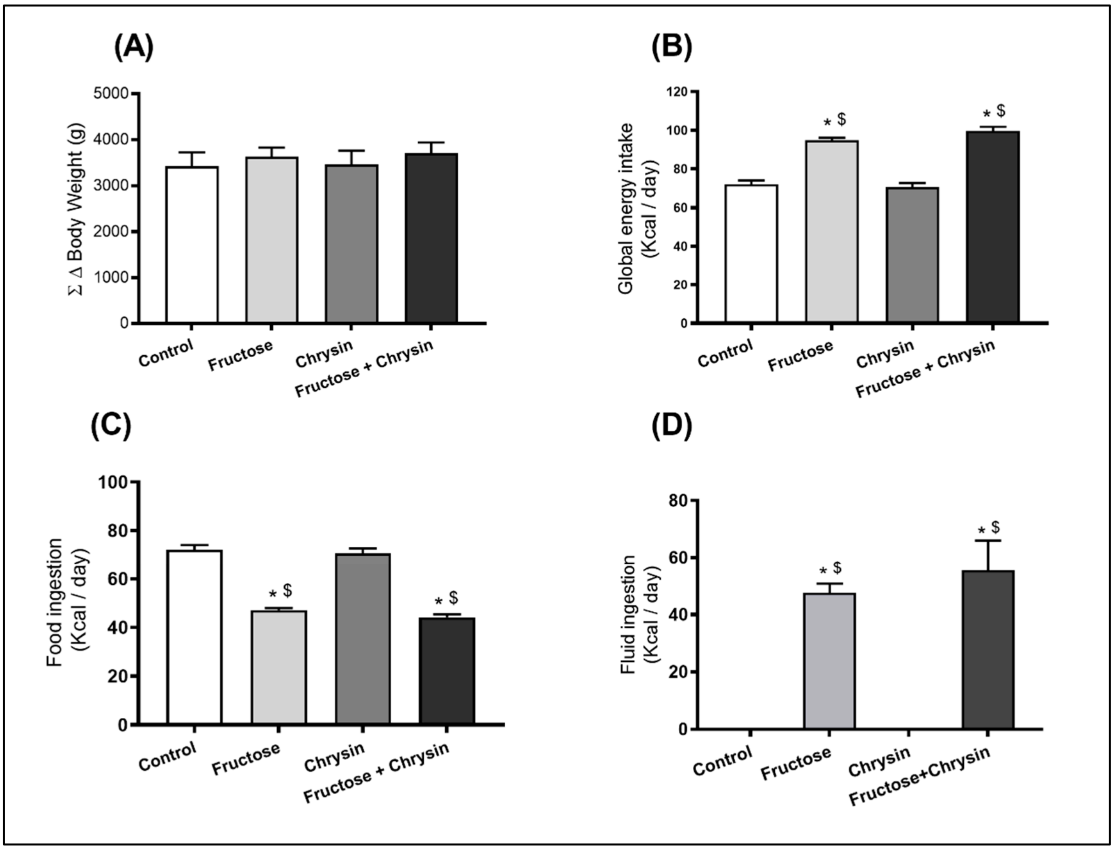

Figure S1. Variation in rat body weight and energy intake (global, from food and from fluid) during the 18 weeks of treatment. (**A**) Σ of the variation in body weight during the treatment period; **(B)** Global (total) energy intake during the treatment period; **(C)** Energy intake from food during the treatment period; **(D)** Energy intake from fluid during the treatment period. Results are expressed as arithmetic means ± SEM (n = 6 per group). *$p < 0.05$ vs control; $ vs. chrysin. Adapted from [1].

Reference

1. Andrade, N.; Andrade, S.; Silva, C.; Rodrigues, I.; Guardao, L.; Guimaraes, J.T.; Keating, E.; Martel, F. Chronic consumption of the dietary polyphenol chrysin attenuates metabolic disease in fructose-fed rats. *Eur. J. Nutr.* **2020**, *59*, 151–165. https://doi.org/10.1007/s00394-019-01895-9.
